# Estrogen Receptor Functions and Pathways at the Vascular Immune Interface

**DOI:** 10.3390/ijms22084254

**Published:** 2021-04-20

**Authors:** Aida Dama, Chiara Baggio, Carlotta Boscaro, Mattia Albiero, Andrea Cignarella

**Affiliations:** 1 Department of Medicine, University of Padova, 35128 Padova, Italy; aida.dama@unipd.it (A.D.); mattia.albiero@gmail.com (M.A.); 2 Department of Pharmaceutical and Pharmacological Sciences, University of Padova, 35128 Padova, Italy; chiara.baggio.5@phd.unipd.it (C.B.); carlotta.boscaro@unipd.it (C.B.); 3 Venetian Institute of Molecular Medicine, 35129 Padova, Italy

**Keywords:** estrogens, estrogen receptors, gender differences, endothelium, monocytes, macrophages

## Abstract

Estrogen receptor (ER) activity mediates multiple physiological processes in the cardiovascular system. ERα and ERβ are ligand-activated transcription factors of the nuclear hormone receptor superfamily, while the G protein-coupled estrogen receptor (GPER) mediates estrogenic signals by modulating non-nuclear second messengers, including activation of the MAP kinase signaling cascade. Membrane localizations of ERs are generally associated with rapid, non-genomic effects while nuclear localizations are associated with nuclear activities/transcriptional modulation of target genes. Gender dependence of endothelial biology, either through the action of sex hormones or sex chromosome-related factors, is becoming increasingly evident. Accordingly, cardiometabolic risk increases as women transition to menopause. Estrogen pathways control angiogenesis progression through complex mechanisms. The classic ERs have been acknowledged to function in mediating estrogen effects on glucose metabolism, but 17β-estradiol also rapidly promotes endothelial glycolysis by increasing glucose transporter 1 (GLUT1) and 6-phosphofructo-2-kinase/fructose-2,6-biphosphatase 3 (PFKFB3) levels through GPER-dependent mechanisms. Estrogens alter monocyte and macrophage phenotype(s), and induce effects on other estrogen-responsive cell lineages (e.g., secretion of cytokines/chemokines/growth factors) that impact macrophage function. The pharmacological modulation of ERs for therapeutic purposes, however, is particularly challenging due to the lack of ER subtype selectivity of currently used agents. Identifying the determinants of biological responses to estrogenic agents at the vascular immune interface and developing targeted pharmacological interventions may result in novel improved therapeutic solutions.

## 1. The Vascular Immune Interface

Maintenance of vascular homeostasis is critical in both physiology and pathology, and the endothelium plays a significant role in vascular function [1]. Among other risk factors, low-density lipoprotein cholesterol (LDL-C) plays a central role in the development of endothelial dysfunction and atherosclerotic plaques. LDL-C, and probably other lipoproteins such as the small dense LDL particles and lipoprotein (a), traverse endothelial cells and undergo oxidative modification by reactive oxygen species. Once oxidized, these lipoproteins can promote atherosclerosis through several different mechanisms. First, oxidized lipoproteins facilitate monocyte recruitment. Second, oxidized lipoproteins may stimulate the expression of adhesion molecules of the vascular endothelium, promoting the adhesion of these monocytes to the vascular wall, one of the first steps in plaque development [2,3]. Third, these monocytes can then enter the vascular intima where they take up oxidized lipoproteins via scavenger receptors, converting them to foam cells. Finally, the oxidized lipoproteins can directly damage endothelial cells, further enhancing the rate of LDL penetration into the vessel [2,3].

Accumulation of inflammatory cells is a critical step in the development and progression of atherosclerotic lesions [4]. Monocytes are released from bone marrow into the circulatory system and they reach target tissues in response to injury, where they differentiate into mature macrophages. In the vessel wall, monocyte-derived macrophages are involved in the initiation of atherogenesis and formation of fatty streaks [5]. The focal attachment of monocytes to the endothelium and their subsequent transendothelial migration are important components for atherosclerotic lesion formation and progression [4,5]. Pro-inflammatory leukocytes are recruited to the site of atherosclerotic plaque development through interaction with several adhesion molecules expressed by endothelial cells. Through several mechanisms, monocytes perpetuate the inflammatory response to noxious stimuli, form foam cells and produce locally released cytokines (interleukin (IL)-6, TNF-α), which are important for plaque formation [6]. Subsets of activated monocytes are also necessary for the initiation and modulation of the immune response, mainly through activation of nuclear factor kappa B (NF-κB)-related transcription, leading to the production and secretion of proinflammatory signaling mediators and cytokines [7,8].

In summary, atherosclerosis can be considered as both a lipid metabolism disorder and a chronic inflammatory disease. Macrophages play a central role in atherogenesis through the accumulation of cholesterol and the production of inflammatory mediators and cytokines. This series of events occurs at the vascular immune interface, which represents a critical site for targeted pharmacological intervention [2,9].

## 2. Cardiovascular Risk in Women

Cardiometabolic risk increases with progressing age in both sexes and especially with menopausal transition in women. This increased risk coincides with the reproductive hormone loss that occurs as women transition to menopause. Women are protected from atherosclerosis until menopause, a finding attributed to the shielding effect of estrogens. 17β-estradiol (E2) is the major circulating estrogen in pre-menopausal females; several lines of evidence suggest that E2 has protective effects on the cardiovascular system, but the molecular mechanisms remain partially unknown [10,11]. Estrogens have been shown to slow down the development of atherosclerosis both in animal models and in humans [12]. By contrast, postmenopausal decline of estrogen production along with a variety of sex-specific risk factors is believed to be responsible for the increased incidence of cardiovascular disease in women following menopause [13,14]. Cardiovascular disorders are associated with endothelial dysfunction and the activation of the monocyte-macrophage system [15,16]. As discussed below, postmenopausal estrogen loss is also associated with impaired alternative activation in macrophages, which may contribute to the worsening of cardiovascular risk profile [17].

## 3. Estrogen Receptors in the Cardiovascular System

Estrogens exert both rapid and long-term actions through their binding with estrogen receptors (ERs). ERs are ligand-inducible transcription factors and are members of the nuclear hormone receptor superfamily. Several ERs have been identified: the nuclear subtypes, ERα and ERβ, and the transmembrane G-protein-coupled receptor 30/G-protein estrogen receptor (GPER) [18]. ERα and ERβ mainly act as transcription factors responsible for many genomic effects, modulating gene expression by direct binding to DNA at specific estrogen response elements. Splice variants of the full-length ERα (ERα66) including ERα36 and ERα46 have been identified in different cell types including human macrophages [19,20] and appear to mediate rapid anti-inflammatory estrogen actions. There is evidence for ERβ splice variants in peripheral blood mononuclear cells (PBMC) [21] but their functional role has not been investigated. GPER is mainly involved in mediating rapid intracellular responses induced by E2 [22]. Membrane localizations of sex steroid receptors are generally associated with rapid, non-genomic effects, while intracellular localizations are associated with nuclear/transcriptional activities.

GPER is a newly discovered 7-pass transmembrane receptor that mediates many of the acute as well as chronic effects of E2. GPER mediates estrogenic signals by modulating non-nuclear second messengers, including activation of the mitogen-activated protein kinases (MAPK) signaling cascade [23]. Since its recognition as an estrogen receptor about 15 years ago, its roles in the cardiovascular system have been increasingly recognized. For instance, chronic in vivo GPER activation mimics the antihypertensive effects of estradiol [24]. GPER moderates many Ca^2+^-dependent activities that control cardiovascular pressor responses through a feed-forward loop in which GPER mediates the actions of E2 [25].

The functional role of ER subtypes has been investigated in vivo. For instance, experimental evidence indicates that targeted deletion of the *Esr1* gene encoding for ERα results in several abnormalities, including tissue inflammation and insulin resistance [26]. Mice deficient in the *Esr2* gene encoding for ERβ display increased systemic arterial blood pressure [27]. Increased vasoconstrictor tone has been observed in *Gper*-deficient mice [28]. We previously reported that rapid relaxion of precontracted arterial tissue is triggered by ERα- but not ERβ-selective agonists [29]. However, dissection of specific estrogen signaling mechanisms is complicated by tissue specific estrogen regulation of transcription, membrane-delimited signaling that synergizes in mediating transcriptional changes and ligand-independent ER regulation of transcription [18]. In addition, cross talk between ERs has been reported for a number of endpoints. For example, E2-induced NO release is substantially reduced in the presence of the GPER-selective antagonist G36, suggesting that both ERα and GPER are involved in this process [30,31].

## 4. Sex Differences and Estrogenic Pathways Regulate Endothelial Angiogenesis

Gender dependence of endothelial biology, either through the action of sex hormones or sex chromosome-related factors, is becoming increasingly evident. Sex genotype and exposure to sexual hormones are relevant in angiogenesis outcomes. The hormonal microenvironment (i.e., estrogen exposure) and sex chromosomes modulate human umbilical vein endothelial cell (HUVEC) functional phenotypes and signaling involved in angiogenesis, demonstrating that the two features are important in conditioning the angiogenic response [32,33].

E2 stimulates endothelial cell proliferation in vitro [34] and in vivo [34,35,36], and inhibits spontaneous as well as TNF-α-induced apoptosis [37,38]. Furthermore, E2 enhances adhesion of HUVECs to matrix proteins and increases cell migration, thus promoting angiogenesis [34,39]. The mechanisms responsible for the proangiogenic effect of E2 have been widely investigated and appear to be mediated at least in part by ERα activation [40]. In particular, E2 regulates actin remodeling and cell movement in HUVECs through the recruitment of focal adhesion kinase (FAK) [40]. Analysis of ERα knockout mice suggests that functional ERs are essential for the augmentation of basic fibroblast growth factor-induced angiogenesis by exogenous E2 [41].

Estrogenic pathways control angiogenesis through complex mechanisms [42]. An emerging regulatory mechanism is suggested by the observation that angiogenic signaling pathways converge onto metabolism [43]. The classic ERs have been acknowledged to function in mediating estrogen effects on glucose metabolism [44]. In addition, E2 rapidly promotes glycolysis in healthy endothelial cells by increasing glucose transporter 1 (GLUT1) and 6-phosphofructo-2-kinase/fructose-2,6-biphosphatase 3 (PFKFB3) levels. By interacting with GPER, E2 and the GPER agonist G1 enhance endothelial PFKFB3 stability and tubulogenesis by increasing deubiquitinase USP19 levels, thereby reducing PFKFB3 ubiquitination and proteasomal degradation. This involves a novel mechanism of estrogenic regulation of PFKFB3 mediated by GPER. E2 and G1 also increase endothelial GLUT1 protein expression via GPER through different mechanisms [45,46]. These findings suggest that ERs represent potential targets to afford selective modulation of endothelial function and angiogenesis.

Angiogenesis is an in vivo phenomenon of multiple cell types: both endothelial and vascular smooth muscle cells can show the network formation in vitro. Targeting these pathways with ER selective agents might be a more rewarding strategy than current angiogenesis inhibitors, which induce remarkable cardiovascular side effects. Clinical aspects/complications risk that come with widespread angiogenesis blockade include stroke, pulmonary embolism and renal failure [47]. In view of the role of ER pathways at the crossroads of progression of certain cancer types and control of vascular function, ERs may represent promising targets for selective pharmacological modulation and personalized medicine.

## 5. Estrogenic Pathways in the Monocyte-Macrophage System

### 5.1. Patterns of Estrogen Receptor Expression in Monocytes and Macrophages

ER expression has been investigated in human monocytes and macrophages. These cells express all ERs. In primary monocyte-derived human macrophages and monocytic THP-1 cells, transcripts for both ERα and ERβ were identified [17,19,20,48]. In primary human monocytes, ERα36 and GPER appear to be predominant [20]. Expression of ERα is greater than ERβ in monocytes, while macrophages express higher levels of ERα and lower levels of ERβ than monocytes. Full length ERα66 and the ERα46 splice variant are expressed in primary human monocytes and macrophages [19]. E2 induces ERα46 in macrophages, but has no effect on ERα expression in monocytes. Monocytes and macrophages also differ in the pattern of ERα66 and ERα46 expression: monocytes express equivalent levels of the two proteins, while in macrophages ERα46 is more highly expressed than ERα66 [19]. ERβ transcript and ERβ protein are not regulated by estrogen levels, suggesting a lack of autoregulation [49]. Many immune effects attributed to E2 in monocytes and macrophages are thought to be mediated through ERα and not ERβ [19,49,50]. In fact, ERα and ERβ target genes differ substantially. Campesi and colleagues [51] assessed the ability of lipopolysaccharide (LPS) to modulate in a sex-specific manner the expression and activation status of ERα and ERβ in blood monocyte-derived macrophages. In basal conditions, ERα and ERβ were significantly higher in female than in male monocytes-derived macrophages. LPS upregulated ERα and its phosphorylation in both sexes, with a significantly higher effect observed in male macrophages, and downregulated ERβ level in female macrophages only [51]. Also, GPER is expressed in human monocytes and macrophages, where it mediates E2 anti-inflammatory actions [20].

### 5.2. Estrogenic Pathways at the Vascular Immune Interface

The role of monocytes and the effects of estrogen/ER pathways on these cells are especially relevant in atherogenesis. E2 induces a particularly robust modulatory effect on monocyte chemotaxis by reducing expression of monocyte chemotactic protein-1 (MCP-1, also known as CCL2), which results in decreased macrophage recruitment to the vessel wall [52]. E2 directly targets monocytes and inhibits monocyte adhesion under flow conditions [53]. Prior studies have shown that estrogens might indirectly affect monocyte adhesion by inhibiting adhesion molecule expression on the endothelial surface [50,53,54].

As noted in Section 1 above, some LDL enters the arterial wall, where it undergoes modification (e.g., oxidation). Modified LDL induces expression of MCP-1, which recruits monocytes into the artery wall and stimulates their differentiation into macrophages. Macrophage uptake of the modified LDL results in formation of foam cells, the hallmark cell of atherosclerosis. Estrogens have a protective effect in the arterial wall through enhanced cellular cholesteryl ester hydrolysis and reduced LDL accumulation and degradation, processes dependent on foam cells (or lipid-loaded macrophages). Estrogens also inhibit oxidation of LDL by macrophages and can induce a direct antioxidative effect, thus reducing macrophage activation by oxidized LDL and preventing atherosclerosis progression [12,48,49,50,55]. Impaired ERα action in macrophages is causal for the development of aspects of the metabolic syndrome and increased atherosclerotic lesion formation in female mice [56], consistent with the notion that the atheroprotective effects of estradiol are largely mediated by ERα [57].

Estrogens may also affect expression of other members of the nuclear receptor superfamily with a relevant role in atherosclerosis. In particular, the liver X receptor (LXR) is a sterol sensor that regulates intracellular cholesterol homeostasis and macrophage cholesterol efflux [58]. LXRs exert atheroprotective effects in the macrophage: in addition to regulating cholesterol metabolism, LXRs are also negative regulators of macrophage inflammatory gene responses [59]. Kramer et al. [48] reported that estrogen removal induces a significant decrease in the transcript levels of LXRα. As discussed in Section 6 below, endogenous LXR ligands can also activate ERs. This suggests the occurrence of nuclear receptor cross-talk in macrophages [49,60], which warrants further investigation.

As described in more detail in Section 5.3 below, estrogens can alter macrophage phenotype(s) and function. However, beyond the direct effects on macrophages, there are effects of E2 on other estrogen-responsive cell lineages that can impact macrophage function (e.g., secretion of a variety of cytokines/chemokines/growth factors). For instance, monocyte/macrophage function is controlled by lymphocytes [61,62]; estrogenic modulation of these parameters has been found in humans and animals. Lymphocytes (as monocyte/macrophage function regulators) are also target for estrogens and express ERs, which may regulate, for example, IL-10 and IL-17 release [63]. Estrogen-mediated protection from inflammation also depends on the presence of B-cells [64]. Sex differences in CD4^+^ T cell and monocyte proportions are relevant and affected by ageing [65]. Of note, sex hormones act as epigenetic modifiers in innate immune cells [66]. It has been reported that the CD4+/CD8+ ratio is associated with DNA methylation in postmenopausal women [67]. Therefore, ER action on epigenetic reprogramming plays a critical role in lymphocytes and may contribute to low-grade chronic inflammation as linked to menopause.

### 5.3. Estrogens: Regulators of the Immune Function of the Monocyte-Macrophage System

ERα has anti-inflammatory actions that ERβ does not possess, consistent with the notion that overall ERα has greater protective effects than ERβ. In addition to ERα and ERβ, estrogen can activate GPER, which is also found in macrophages. The potential role of GPER in immune cells and metabolic disease has been reviewed recently [68]. Macrophages are instructed by estrogens through receptor-mediated mechanisms of action to enable faster resolution of the inflammatory response and proper tissue remodeling. ERα null mutation in myeloid cells is an essential tool to dissect the direct versus indirect effects of E2 in macrophages [56], and reveal the contribution of ERα in maintaining key macrophage functions such as oxidative metabolism, phagocytosis, cholesterol uptake and phenotypic activation [56,69,70].

Estrogens are known modulators of monocyte/macrophage functions; however, the underlying mechanism are still under investigation [20,49,50]. Several studies have shown that E2 acts as a regulator of the immune function of the monocyte-macrophage system, especially regarding the production of cytokines: their effects on the monocyte-macrophage system are primarily repressive [70,71,72,73]. Most of these effects are mediated by repression of gene expression for pro-inflammatory cytokines or other inflammatory mediators by ER-dependent or nongenomic pathways. The ER-dependent mechanisms mostly involve regulation of activity of the NF-κB pathway for transcriptional regulation of cytokines or other mediator genes. However, conflicting results have been reported from studies investigating the effects of estrogens on macrophage effector functions [20,50,69]. The estrogen-ER complex has been reported to inhibit binding of the NF-κB complex to regulatory areas of target genes, or to prevent nuclear translocation and transcriptional activation of the *TNF-α* gene [74]. IL-6 is one of the main cytokines involved in chronic inflammation-related monocyte functions. E2 is known to inhibit expression of TNFα, IL-1 and IL-6. However, chronic exposure of murine macrophages to E2 in vivo increases production of pro-inflammatory cytokines [69]: in this regard, the literature is discordant with E2 enhancing or inhibiting secretion of TNF and IL-1β likely related to the duration of estrogen exposure and experimental design [72]. Long-term in vivo exposure to estrogens from endogenous or exogenous origin enhances the LPS-induced transcription of proinflammatory cytokines (IL-12, TNF-α) by microglial cells through ERα-dependent mechanisms [75]. In one study the anti-inflammatory effect of short-term in vitro exposure to E2 was reported in murine resident peritoneal macrophages [76], but chronic administration of E2 to ovariectomized female mice markedly increases the expression of numerous inflammatory cytokines and NO to LPS activation ex vivo [69]. In vitro pre-treatment with E2 of human macrophages inhibits the NF-κB signaling pathway and the production of TNF-α induced by LPS [20,76]. A significant increase in LPS-induced TNF-α release has been reported in ERα-deficient macrophages, suggesting a prominent role of ERα in mediating the anti-inflammatory effects of estrogen. The deletion of ERα in hematopoietic cells in mice also causes an inability to induce the alternative phenotype in IL-4-stimulated macrophages as well as high levels of inflammation and insulin resistance, suggesting that ERα is involved in the control of inflammation [56,77]. Expression of the proinflammatory mediator IL-8/CXC-motif ligand8 (CXCL8) is also decreased by E2 in LPS-challenged monocytes, providing evidence of a direct correlation between ERα expression levels and suppression of LPS-induced IL-8 secretion [78].

**Figure 1:**
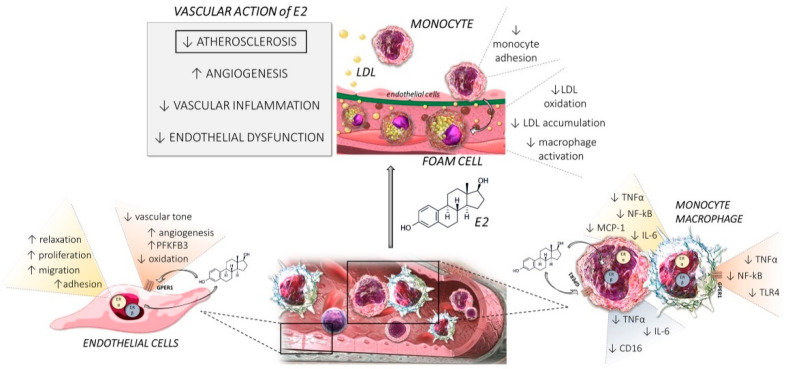
Estrogen/ER pathways induce protective effects in the vessel wall with specific actions on endothelial cells and monocytes/macrophages through various pathways. E2 attenuates inflammation by regulating the induction of chemokines and cytokines at the vascular immune interface that are mediated largely by ERα activation. The interaction between endothelial cells and monocytes/macrophages is relevant in multiple disease settings such as atherosclerosis.

E2 can significantly influence CD16 expression, a receptor mediating autoimmune disease symptoms, and alter monocytic cytokine release after CD16 receptor activation. E2 reduces CD16 expression and decreases TNF-α and IL-1β release after CD16 stimulation [79]. Kramer et al. reported that CD16 expression can be altered by ER activity and that ERα can associate with a region in the CD16 promoter involved in transcript production [80]. ERβ agonist treatment also reduces CD16 expression in macrophages [80] and attenuates the decrease in macrophage IL-6 and TNF-α production by splenic macrophages after trauma hemorrhage with no effect on the activation of MAPKs and NF-κB [81]. Xing et al. showed the ability of selective ERβ activation to inhibit expression of inflammatory mediators [82]. The main effects of E2 on monocyte-macrophages and endothelial cells are depicted in Figure 1.

Another major pathway of estrogen regulation of the monocyte-macrophage system is stimulation of production of members of the immunosuppressive transforming growth factor (TGF)-β family [50]. Functional effects of the endocrine disruptor bisphenol A in inducing TNF-α and IL-6 production, and inhibiting TGF-β and IL-10 production via ERα/ERβ/ERK/NF-κB signaling have been reported in human THP-1 macrophages [83]. Using a transcriptomic approach, Pepe et al. [84] obtained a comprehensive list of genes that are differentially expressed in peritoneal macrophages in response to physiological levels of E2 injected in intact female mice. They reported that E2 promotes an anti-inflammatory and pro-resolving macrophage phenotype, which converges on the induction of genes related to macrophage alternative activation and on IL-10 expression in vivo [84].

### 5.4. Estrogens: Regulators of Macrophage Immunophenotypes

Macrophages can be stimulated to distinct functional phenotypes: M1 (classically activated) macrophages, M2 (alternatively activated) macrophages and tumor-associated macrophages based on surface markers and cytokine profiles. M1 and M2, however, represent the two extremes in a much more complex phenotype series [85]. There is evidence for an age-relationship of estrogen-macrophage polarization, since cord mononuclear cells respond and post-menopausal monocytes do not [86,87,88]. In this regard, E2 (and progesterone) impair the response of human cord blood mononuclear cells exposed to microbial products, suggesting that maternal hormones regulate neonatal immune responses. Newborn monocytes are more sensitive to the effects of E2 and progesterone than adult peripheral blood mononuclear cells and monocytes [89].

Much earlier on in life, monocytes contribute to atherosclerotic disease by abnormally trafficking to the vessel wall. By the time menopause arrives, atherosclerosis may be already established. There is an acceleration of atherosclerosis post-menopause and, in part, this is secondary to adverse changes in the serum lipid profile with an increase in LDL-C and decrease in HDL-C. Gene expression changes have been reported comparing the whole heart gene expression profile for aged rats with and without estrogen replacement or with late estrogen replacement, which induced paradoxically a pro-inflammatory set of gene changes [90]. This is consistent with the notion that menopausal hormone therapy may be beneficial for the prevention of cardiovascular disease in post-menopausal women when started less than 10 years but not more than 10 years after the menopause, according to a rigorous Cochrane meta-analysis [91]. We have reported that E2 treatment in vitro prevents LPS-IFNγ-induced downregulation of alternative activation markers and cytokine production [17]. There is a differential response of post-menopausal versus pre-menopausal macrophages with a blunted response to M2 stimuli in the latter, suggesting that this affects the post-menopausal woman’s cardiovascular risk profile.

Macrophage activation is also functionally relevant in tissues other than the vascular wall. Macrophages cause activation of several intracellular pathways in breast cancer cells of which c-Src, protein kinase C and MAPK are essential for loss of ERα expression [92]. Thus, it is possible that one subtype of estrogen receptor (GPER) could actually downregulate the other type (ERα). Activation of MAPK by GPER (which can cause loss of ERα) can be described as a feedback inhibition loop for estrogen activation of macrophages and monocytes, and could regulate the balance between M1 and M2 macrophage development. Agents that interfere with ER signaling such as the endocrine disruptor bisphenol A upregulate M1 type responses in the liver of wild-type mice [93].

Further research studies are needed to unravel relevant signaling cascades leading to macrophage polarization and the role of estrogen and other second messengers in it.

## 6. A Possible Role for Estrogens in Constraining Myelopoiesis and Cardiovascular Risk

Hematopoietic stem cells (HSCs) are pluripotent stem cells that produce mature blood cells throughout life, nested in a specialized microenvironment in the adult bone marrow defined “niche” [94]. The niche is as important as the stem cells themselves in regulating their self-renewal and differentiation [95]. ERα is expressed both in osteoblasts, which are a component of the niche, and in different subset of hematopoietic progenitors, even though they lack the expression of ERβ [96,97]. ERα is dispensable for steady-state hematopoiesis [97], but data support the idea that E2 could instruct the differentiation of HSC. Indeed, E2 regulates HSCs self-renewal during pregnancy [97] and improves hematopoietic recovery and regeneration after transplantation and irradiation [97]. Diabetes is associated with a myeloid-skewed differentiation of bone marrow HSCs, termed myelopoiesis [98], which is linked to profound alteration of the homeostasis of the bone marrow [99]. Myelopoiesis is driven by the activation of common myeloid progenitors by neutrophils-released S100A8/9 or macrophage-derived IL-1β [98,100]. The increased amount of pro-inflammatory monocytes and neutrophils released by the bone marrow fuels the progression of atherosclerosis [98] and ultimately contributes to low-grade inflammation and cardiovascular risk. Neutrophils-to-lymphocyte ratio is a marker of systemic inflammation and myelopoiesis, and has been found to be a strong predictor of mortality and cardiovascular risk, while being increased in the elderly and in males [101]. Therefore, it is intriguing to speculate that the effects of estrogens on HSCs might also impinge on restraining the onset of myelopoiesis to explain the protective effects on the cardiovascular system. Some early reports, indeed, showed that E2 reduces myeloid differentiation of HSCs [102,103,104] and described its role in B lymphocyte development [105]. On the other hand, E2 could directly stimulate myeloid differentiation of HSCs in vitro [106]. As mentioned above, HSCs are entangled in specialized niches that supply metabolic support and differentiation stimuli [94]. E2 is known to modulate bone turnover by affecting osteoblasts and osteoclasts, but its effects on these cells as components of the hematopoietic endosteal niche are unknown. Similarly, endothelial cells are essential in regulating HSCs in the perivascular niche [94], but the possible effects of E2 on bone marrow endothelial cells are still unknown. Indeed, E2 can modulate endothelial cell metabolism through GPER [45,46] and this effect could be exploited to modulate the endothelial cell-HSC cross-talk [107], restrain myelopoiesis and ultimately improve cardiovascular health.

## 7. Intricacies of ER Pharmacological Modulation

As noted in Section 3 above, the traditional ERs (ERα and ERβ) are predominantly nuclear-localized proteins, and classically mediate their effects as transcription factors [108]. GPER is a 7-transmembrane GPCR that activates multiple cellular pathways including calcium mobilization, ERK and PI3K via transactivation of the EGF-R [109]. As E2 binds and activates all three ERs (ERα, ERβ and GPER), selective ligands (agonists and antagonists) are needed to unravel and exploit the functional roles of the individual receptors, particularly GPER [110]. However, diverse ER ligands including phytoestrogens (e.g., genistein), xenoestrogens (e.g., bisphenol A), the “ERα-selective” PPT and therapeutic anti-estrogens (e.g., tamoxifen, fulvestrant, raloxifene) act as GPER agonists [111]. Selective estrogen receptor modulators (SERMs) such as tamoxifen and raloxifene provide some degree of tissue selectivity. These agents have been reported to affect the monocyte-macrophage system [50]: for example, raloxifene prevents LDL oxidation and the formation of tyrosyl radicals by myeloperoxidase [112], as well as caspase-3 dependent apoptosis induced by TNF-α in carotid artery endothelial cells [113]. More recently, bazedoxifene has been reported to protect HUVECs from TNF-α-induced inflammatory damage by targeting CD40 [114]. However, currently used SERMs do not display remarkable ER subtype-specificity. Thus, none of currently used endogenous or synthetic ER-targeting agents affords ER subtype selectivity.

We viewed the development of ER subtype-selective therapeutic agents, rather than experimental tools, as an unmet pharmacological need already 15 years ago [115]. Later on, we reported that systemic treatment with the ERα agonist propylpyrazoletriol (PPT) provides cardiovascular protection without undesired ERα-mediated uterine activation in rodents [116], but a contribution of GPER to PPT effects cannot be ruled out. It is encouraging that a GPER-selective agonist is poised to start clinical development following successful preclinical testing [117]. Another pharmacological challenge in the ER field is the development of agents that uncouple nuclear and membrane ERα activation. Estetrol (E4) has shown such a pattern [118]: this agent is less potent than E2 and shows some tissue selectivity as it induces limited effects on the liver [119]. E4 is under clinical development for a few indications including contraception and breast cancer [120,121].

Adding further complexity to the field, nonestrogenic ligands are known to modulate ER activity. Several oxysterols including 27-hydroxycholesterol (27HC) not only activate LXR but also display estrogenic activity [122]. Female murine bone marrow-derived macrophages (BMDMs) show higher ERα expression with respect to male BMDMs. Because ERα and ERβ target genes differ substantially, the binding of ligands (such as 27HC) to different ER subtypes may impact the fate of the inflammatory signaling [123]. The inflammatory effects of 27HC in murine BMDMs are sex-opposed only in the presence of E2, indicating a key role for estrogen in the 27HC-induced effect on inflammation [123]. This observation is in line with previous findings of Umetani et al. [124], who linked 27HC to estrogen by identifying 27HC as the first endogenous SERM. Moreover, the angiotensin AT_1_ receptor antagonist olmesartan suppresses ischemic brain damage, exaggerated in estrogen-deficient rats, at least in part via upregulated expression and phosphorylation of ERα, as well as upregulation of anti-apoptotic genes and of ACE2, resulting in attenuated activation of the renin-angiotensin system after ischemia [125]. These molecular effects occurred in an E2-independent manner and were blocked by fulvestrant, which acts both as a selective ER degrader and as a GPER agonist. Whether off-target ER pathways contribute to olmesartan therapeutic effects in humans remains to be determined.

## 8. Conclusions

Declining estrogen levels are associated with a variety of disorders such as osteoporosis, neuroinflammatory diseases, vascular wall degeneration, cardiovascular diseases and increase the risk of atherosclerosis. Preclinical and clinical evidence suggests that estrogenic agents interfere with early events in atherogenesis taking place at the vascular immune interface. Here modified lipoproteins trigger endothelial dysfunction as well as the accumulation of cholesterol and the production of inflammatory mediators and cytokines by monocyte-derived macrophages. While pharmacological intervention at this site would be instrumental to prevent atherosclerosis progression, current estrogenic agents lack ER subtype-, cell type- and, at least in part, tissue selectivity, leading to systemic undesired effects. Thus, the development of “smart” estrogenic agents targeting the vascular immune interface would provide a novel treatment solution to reduce cardiovascular risk in women.

## Data Availability

Not applicable.
